# Comparative genomic analysis of Brazilian *Leptospira kirschneri* serogroup Pomona serovar Mozdok

**DOI:** 10.1590/0074-02760160174

**Published:** 2016-07-11

**Authors:** Luisa Z Moreno, Frederico S Kremer, Fabiana Miraglia, Ana P Loureiro, Marcus R Eslabao, Odir A Dellagostin, Walter Lilenbaum, Andrea M Moreno

**Affiliations:** 1 Universidade de São Paulo, Faculdade de Medicina Veterinária e Zootecnia, Laboratório de Epidemiologia Molecular e Resistência a Antimicrobianos, São Paulo, SP, Brasil; 2 Universidade Federal Fluminense, Departamento de Microbiologia e Parasitologia, Laboratório de Bacteriologia Veterinária, Niterói, RJ, Brasil; 3 Universidade Federal de Pelotas, Centro de Desenvolvimento Tecnológico, Pelotas, RS, Brasil

**Keywords:** L. kirschneri, serovar Mozdok, genomics

## Abstract

*Leptospira kirschneri* is one of the pathogenic species of the *Leptospira* genus. Human and animal infection from *L. kirschneri* gained further attention over the last few decades. Here we present the isolation and characterisation of Brazilian *L. kirschneri* serogroup Pomona serovar Mozdok strain M36/05 and the comparative genomic analysis with Brazilian human strain 61H. The M36/05 strain caused pulmonary hemorrhagic lesions in the hamster model, showing high virulence. The studied genomes presented high symmetrical identity and the *in silico* multilocus sequence typing analysis resulted in a new allelic profile (ST101) that so far has only been associated with the Brazilian *L. kirschneri* serogroup Pomona serovar Mozdok strains. Considering the environmental conditions and high genomic similarity observed between strains, we suggest the existence of a Brazilian *L. kirschneri* serogroup Pomona serovar Mozdok lineage that could represent a high public health risk; further studies are necessary to confirm the lineage significance and distribution.

Leptospirosis is an emerging worldwide zoonosis which is caused by spirochetes of the *Leptospira* genus (Levett 2001). To date, the genus comprises 11 pathogenic species (Bourhy et al. 2014) of which *Leptospira interrogans* is the most associated with human infection. Nevertheless, *L. kirschneri* infection has gained further attention over the last few decades.

Masuzawa et al. (2006) reported one of the first cases of *L. kirschneri* human infection by direct contact with southern flying squirrels imported from the United States; two Japanese workers were infected by *L. kirschneri* serovar Grippotyphosa from the imported American exotic pets. The *L. kirschneri* serogroup Pomona serovar Mozdok has also been related to human infection in Cuba (Obregón et al. 2007). In Europe, the serovar Mozdok is described as endemic in wild rodents (Majetic et al. 2014) and has also been associated with canine leptospirosis (Renaud et al. 2013).

In Brazil, *L. kirschneri* serogroup Pomona serovar Mozdok has recently been described as the causative agent of human and canine infection in different time points (Cunha et al. 2016). Here we present the isolation and characterisation of *L. kirschneri* serogroup Pomona serovar Mozdok strain M36/05 and the comparative genomic analysis with previously described Brazilian *L. kirschneri* serogroup Pomona serovar Mozdok human strain 61H.

The M36/05 strain was isolated from the kidney of a captured urban black rat (*Rattus rattus*) in Suzano, metropolitan region of São Paulo State, Brazil, in 2005. For isolation, 5 g of kidney sample was collected and homogenised in 50 mL of Sorensen saline, and 100 µL aliquots of 10^-1^ to 10^-3^ dilutions were inoculated into duplicate tubes containing EMJH (DIFCO, USA) enriched with 15% rabbit serum, 5-fluorouracil and nalidixic acid. Once isolated, the strain was stored in Fletcher’s medium (DIFCO/USA), enriched with 15% rabbit serum and maintained in EMJH broth (DIFCO/USA) at 30ºC, as part of the *Leptospira* collection of the Laboratory of Bacterial Zoonosis - University of São Paulo.

Serogrouping was performed at the Laboratory of Veterinary Bacteriology - Fluminense Federal University. The isolate was subjected to microscopic agglutination test (MAT) using a panel of polyclonal rabbit antisera of 32 reference serovars representing the 24 known serogroups (provided by Royal Tropical Institute - KIT, Amsterdam). The M36/05 strain presented high agglutination rates with serogroup Pomona antisera which was then identified as the strain presumptive serogroup (Dikken & Kmety 1978).

To evaluate the strain virulence, five Golden Syrian hamsters (*Mesocricetus auratus*) were infected with M36/05 strain (10^8^ leptospires) through intraperitoneal route. The animal experiment was conducted with the approval of the Ethics Committee from the School of Veterinary Medicine and Animal Science - University of São Paulo (2244/2011). Clinical symptoms were checked daily. Within five days post-infection, the M36/05 strain caused lethal leptospirosis and the infected hamsters were euthanised. The inoculated animals developed acute infection characterised mainly by haemorrhagic pulmonary lesions (Fig. 1).

**Fig. 1:**
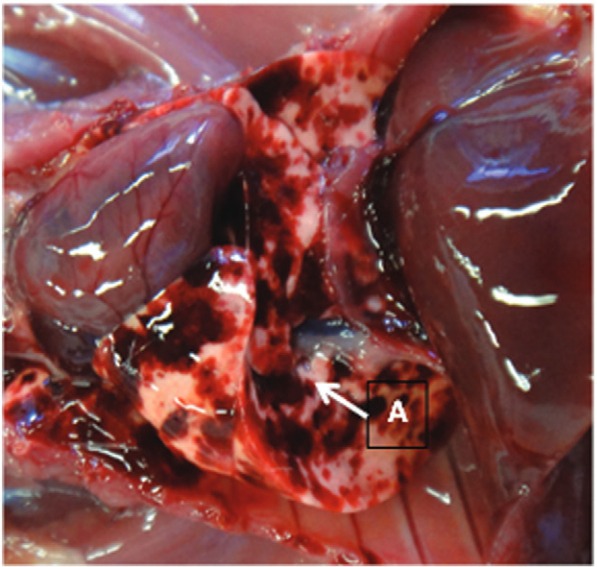
: macroscopic lesions observed in respiratory tract of M36/05 infected hamsters. (A) The main feature was haemorrhagic pulmonary lesions.

Genomic DNA was extracted and purified with illustra^™^ bacteria genomicPrep Mini Spin Kit (GE Healthcare do Brasil Ltda, São Paulo, Brazil) and used for paired-end library preparation with Nextera^™^ DNA Sample Prep Kit (Illumina^®^) and sequencing through Illumina^®^ Miseq platform. The *de novo* assembly was performed with Geneious 8.1.8 (Biomatters Ltd, Auckland, New Zealand) and CLC Main Workbench 7.5.1 (CLC Bio, Denmark) and resulted in 44 scaffolds with a N_50_ of 241,247 bp.

The M36/05 draft genome (LLJK00000000) comprises ~4.46 Mb with overall GC content of 35.9%. Automatic genome annotation was performed with NCBI Prokaryotic Genome Annotation Pipeline. The basic annotation features identified in M36/05 strain are summarised in Table. With regard to the virulence genes, M36/05 presents genes encoding the main *Leptospira* virulence factors as lipoproteins and immunoglobulin-like proteins (*ligA*, *ligB, ligC lolC/D*, *lipA, lipL32* and *loa22*) and also flagellar proteins (*flaA* and *flab* subunits, *fliG/F*). In addition, antimicrobial resistance genes were also identified (*tetA*, *ermA*), including efflux pumps (*norM*, *mdtA* and *qacA*).

**TABLE:** Assembly statistics, basic annotation features and sequence types for respective *Leptospira* multilocus sequence typing (MLST) schemes observed for Brazilian *L. kirschneri* serogroup Pomona serovar Mozdok strains

Strain	Assembly statistics					Basic annotation features				*Leptospira* MLST schemes		
		
	Scaffolds	N_50_	Length	CG%		CDS	rRNAs	tRNAs		Ahmed et al. (2006)	Boonsilp et al. (2013)	Varni et al. (2014)
M36/05	44	241,247	4.46 Mb	35.9		3,606	6	37		ST98	ST117	ST101
61H	174	45,311	4.48 Mb	35.9		3,629	5	38		ST98	ST117	ST101

The M36/05 genome was compared to the Brazilian *L. kirschneri* serogroup Pomona serovar Mozdok human strain 61H (JSVJ00000000) through Mauve multiple genome aligner (Darling et al. 2004) and BLAST Ring Image Generator (BRIG) (Alikhan et al. 2011) and presented high symmetrical identity (98.86%) (Fig. 2). Due to the unavailability of a complete *L. kirschneri* reference genome, the chromosomes of the studied genomes were not individualised. The genetic content of strains is highly similar (Fig. 2A) and the few structural differ- ences observed (Fig. 2B) could be due to differences in the applied assembly and ordering methodologies between the draft genomes. The absence of a reference genome for *L. kirschneri* still poses a challenge for assertive assembly and comparative analysis.

**Fig. 2:**
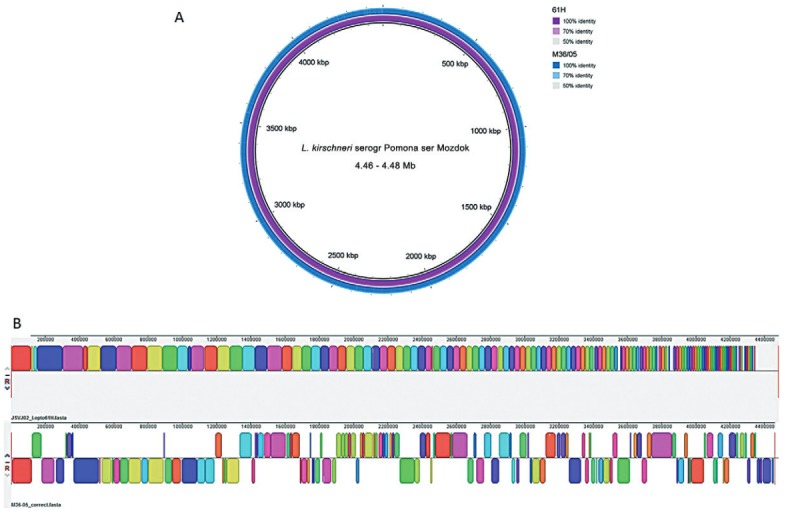
: whole-genome sequencing analysis of Brazilian *Leptospira kirschneri* serogroup Pomona serovar Mozdok strains. (A) BRIG plot displaying genomic similarity; (B) mauve alignment blocks.

The *in silico* multilocus sequence typing (MLST) analysis was performed for the three *Leptospira* MLST protocols available (Table). Both strains presented similar results with ST98 and ST117 for Ahmed et al. (2006) and Boonsilp et al. (2013) protocols, respectively; these sequence types had already been associated with *L. kirschneri* serogroup Pomona serovar Mozdok. For the Varni et al. (2014) protocol, however, both strains presented a new allelic profile (7, 5, 22, 8, 7, 7, 5) which originated a new ST101 that so far has only been associated with the Brazilian *L. kirschneri* serogroup Pomona serovar Mozdok strains.

Considering the geographical and chronological distance between strains, since the 61H strain was isolated from human blood sample from Pelotas, in the metropolitan region of Rio Grande do Sul State, Brazil (~1,380 Km from São Paulo), in 2013 (Cunha et al. 2016), it is possible to infer that *L. kirschneri* serogroup Pomona serovar Mozdok has already been circulating in the southeast and southern regions of Brazil during the last two decades. It has apparently adapted to rodents as a reservoir and presents high virulent potential to humans. *L. kirschneri* serogroup Pomona serovar Mozdok has also been isolated from an asymptomatic dog in Pelotas (Cunha et al. 2016), suggesting that the serovar has already adapted to different reservoir hosts in urban areas.

In view of the environmental conditions and the high genomic similarity observed between strains, this may suggest that they could be a Brazilian *L. kirschneri* serogroup Pomona serovar Mozdok lineage that could represent a high public health risk; further studies are necessary to confirm the lineage significance and distribution. Therefore, it should be included in the *Leptospira* battery of tests of national reference laboratories to enable proper identification and further epidemiological studies.
